# Supplementary material for: WordCommentsAnalyzer: A windows software tool for qualitative research
Source: F1000Res. 2018 Sep 4;7:536. Originally published 2018 May 3. [Version 2] doi: 10.12688/f1000research.14819.2 (PMC6137411; doi:10.12688/f1000research.14819.2)
Supplement: Supplementary file 3 [file f1000research-7-17614-s0002.tgz › b2a79496-4ada-47c4-9682-a99ce867836d.zip!/Eight Iranian Health Journals/Archives of Rehabilitation_48.docx]

© Archives of Rehabilitation. This work is licensed under CC BY-NC 4.0 (https://creativecommons.org/licenses/by-nc/4.0/).

Volume & Issue: دوره 8 - شماره 3

Title: تأثیر یک دوره برنامه تمرینی ترکیبی بر پاسخ‌های همودینامیکی بیماران مرد مبتلا به اختلال عروق کرونر

http://rehabilitationj.uswr.ac.ir/article-1-195-fa.html

هدف: فعالیت بدنی یک نقش پذیرفته شده در توانبخشی قلبی دارد که بیشتر به جهت اثرات مثبت آن بر جریان خون عضله قلب در بیماران عروق کرونر می‌باشد. بهبود سیستم عروق محیطی و عملکرد انقباضی عضله قلب بعنوان سازگاریهای احتمالی پیشنهاد شده‌اند. هدف این مطالعه بررسی اثر تمرینات ترکیبی بر پاسخ‌های همودینامیکی بیماران عروق کرونر می‌باشد. روش بررسی: در این مطالعه تجربی و مداخله‌ای 24 بیمار عروق کرونر که به روش مبتنی بر هدف و لحاظ کردن معیارهای ورود به مطالعه از بین 42 بیمار «جامعه در دسترس» 75-50 ساله انتخاب شده بودند، بطور تصادفی به دو گروه مساوی مداخله و کنترل تقسیم شدند. گروه مداخله در تمرینات ترکیبی به مدت 3 ماه، 3 بار در هفته برای 60 تا 80 دقیقه و در 70% تا 85% ضربان قلب بیشینه و 40% تا 60% یک تکرار بیشینه شرکت کردند. متغیرهای مورد مطالعه در ابتدا و در پایان برنامه با روش امپدانس کاردیوگرافی مورد ارزیابی قرار گرفتند. داده‌های حاصل با استفاده از آزمون تی مورد بررسی قرار گرفت. یافته‌ها: تمرینات موجب کاهش معنی‌دار ضربان قلب و فشار خون استراحتی بیماران شدند. حجم ضربه‌ای از 8±60 به 13±81 میلی لیتر، شاخص ضربه‌ای از 4±33 به 6±44 میلی لیتر بر ضربان بر مترمربع، برون ده قلبی از 1±4 به 1±5 لیتر در دقیقه و شاخص قلبی از 0/5±2 به 0/5±3 لیتر بر دقیقه بر مترمربع افزایش یافت (0/05>P). مقاومت منظم عروقی از 361±1782 به 294±1540 دین بر ثانیه بر سانتیمتر و شاخص مقاومت منظم عروقی از 662±3212 به 558±2751 دین بر ثانیه بر مترمربع بر سانتیمتر کاهش یافت (0/5>P). نتیجه‌گیری: تمرینات ترکیبی از طریق ایجاد سازگاریهای محیطی، مرکزی و قلبی موجب بهبود پاسخ‌های همودینامیکی قلب بیماران عروق کرونر شود.

Title: مقایسه شاخص‌های تنفسی گفتار در کودکان فلج مغزی نوع اسپاستیک وکودکان عادی

http://rehabilitationj.uswr.ac.ir/article-1-196-fa.html

هدف: مقایسه شاخص‌های تنفسی گفتار در مراحل مختلف، از جمله: میانگین حجم جاری، حجم حیاتی و حجم آواسازی در کودکان مبتلا به فلج مغزی اسپاستیک و مقایسه این شاخص‌ها با کودکان طبیعی و بکارگیری میزان این تفاوتها در برنامه‌ریزیهای دقیق‌تر گفتار درمانی، اصلی ترین انگیزه این تحقیق بوده است.   روش بررسی: تحقیق حاضر به روش مقطعی مقایسه‌ای و از نوع مورد–شاهدی است. با بکارگیری روش هدفمند و غیر تصادفی، بیست نفر کودک دختر و پسر فلج مغزی نوع اسپاستیک 12-5 ساله بعنوان بیمار و بیست نفر کودک عادی دختر و پسر با روش همتاسازی و جور کردن به عنوان گروه شاهد انتخاب شدند. بررسی پرونده سلامتی گذشته و آزمونهای اولیه بالینی و پاراکلینیکی مناسب برای همسان‌سازی و لحاظ معیارهای ورود و خروج از تحقیق و همچنین آزمونهای رادیولوژیکی ریه و آزمونهای خونی در جهت رد امکان هر گونه بیماری احتمالی زمینه‌ای ریوی وسیستمیک در هر دو گروه انجام پذیرفت. متغیرهای حجم جاری، حجم حیاتی و حجم آواسازی در گروه‌های نمونه و شاهد با استفاده از دستگاه کامپیوتری رسپیرومتر که ساخت و هنجار شده در مرکز تحقیقات دانشگاه گلاسکو انگلستان است سنجیده شد. داده‌های حاصل با استفاده از آزمون آماری تی مستقل مورد تحلیل قرار گرفت.   یافته‌ها: تفاوت معنی‌داری در متغیرهای میانگین حجم جاری، حجم حیاتی و حجم آواسازی بین گروه‌های مورد و شاهد وجود داشت (0/001>P). بدین صورت که این شاخص‌ها در افراد بیمار بطور متوسط 34% کمتر از افراد سالم بود.   نتیجه‌گیری: بر اساس نتایج این تحقیق، شاخصهای تنفسی گفتار در کودکان فلج مغزی اسپاستیک کاهش محسوسی داشته و این امر باید در برنامه‌ریزی دقیق‌ و مشخص‌ توانبخشی و گفتاردرمانی این کودکان مد نظر بوده و بکار گرفته شود.

Title: بررسی تأثیر خستگی موضعی عضلات دیستال وپروگزیمال در صفحه ساژیتال اندام تحتانی بر میزان وابستگی به بینایی در ثبات پاسچر زنان جوان سالم

http://rehabilitationj.uswr.ac.ir/article-1-198-fa.html

هدف: این تحقیق به منظور بررسی تأثیر خستگی موضعی عضلات دیستال وپروگزیمال در صفحه ساژیتال اندام تحتانی بر میزان وابستگی به بینایی در ثبات پاسچر ایستاده انجام شده است.   روش بررسی: در این مطالعه شبه تجربی (از نوع قبل و بعد)، 25 دختر جوان سالم که به روش نمونه‌گیری غیر احتمالی و ساده از نمونه‌های در دسترس بر اساس معیارهای ورود و خروج انتخاب شده بودند، در طی 2 جلسه با ترتیب تصادفی و فاصله زمانی یک هفته خستگی در عضلات حرکت دهنده مچ و ران آنها در صفحه ساژیتال توسط انقباض ایزوکینتیک ایجاد کردند. از نمونه‌ها خواسته شد تا حد امکان وضعیت ایستاده قائم روی یک پا را حفظ کنند. RMS و انحراف معیار جابه جایی مرکز فشار در مدت 30 ثانیه ارزیابی شده و بعد از 15 ثانیه چشمها بسته می‌شد. از آزمون آماری آنالیز واریانس برای اندازه‌های مکرر جهت بررسی تأثیر سه عامل خستگی، بینایی و موضع خستگی در دوره‌ زمانی 5 ثانیه قبل و بعد از بستن چشم استفاده شد.   یافته‌ها: ‌ اثر خالص هر سه متغیر خستگی، بینایی و موضع خستگی بر متغیرهای وابسته معنی ‌دار بود (P<0/05). آنالیز RMS و انحراف معیار جابه جایی مرکز فشار بیانگر معنی‌دار بودن اثر متقابل خستگی با بینایی و خستگی با موضع خستگی بود، به نحوی که آثار خستگی در موضع پروگزیمال و حالت چشم بسته بزرگتر بود. نتیجه‌گیری: ‌ میزان وابستگی به بینایی جهت کنترل ثبات پاسچر بدنبال خستگی عضلانی افزایش می‌یابد و خستگی موضع پروگزیمال سبب تشدید وابستگی به بینایی جهت کنترل ثبات پاسچر می‌گردد. بر اساس یافته‌های تحقیق حاضر، در تمرینات ثبات پاسچر تأکید بر حس عمقی موضع پروگزیمال اندام تحتانی می‌تواند توصیه شود.

Title: تهیه آزمون جهت بررسی تأثیر شکل و جایگاه نویسه بر خطاهای دیکته در دانش‌آ‌موزان کلاس اول ابتدایی

http://rehabilitationj.uswr.ac.ir/article-1-199-fa.html

هدف: این مطالعه به منظور تهیه آزمونی جهت بررسی تأثیر شکل و جایگاه نویسه بر خطاهای هجی کردن و تعیین روایی و اعتبار آن در پایه اول ابتدایی انجام شد.   روش بررسی: این مطالعه به روش توصیفی و مقطعی و از نوع ابزارسازی و اعتبارسنجی می‌باشد. جمعیت مورد مطالعه دانش‌آ‌موزان کلاس اول ابتدایی (دو کلاس از مدرسه‌‌ دخترانه و دو کلاس از مدرسه‌ پسرانه) از منطقه 8 آموزش و پرورش شهر تهران بودند که به روش غیر تصادفی و ساده انتخاب شدند.   ابتدا کلمات کتاب «بنویسیم» پایه اول ابتدایی بر اساس شکل و جایگاه نویسه مورد بررسی قرارگرفته و تعدادی واژه انتخاب و برای بررسی به صاحب نظران با تخصص در حیطه‌های آسیب شناسی گفتار و زبان، زبان شناسی و معلمان داده شد. بر اسا س نظرات ارسالی 54 واژه از بین سایرین برای بررسی شکل و 39 واژه برای بررسی جایگاه نویسه انتخاب گردید. به منظور بررسی اعتبار (قابلیت تکرارپذیری) آزمون، کلمات یکبار توسط آزمونگر و معلم در طی دو مرحله به تفکیک به دانش‌آ‌موزان یک کلاس دخترانه و یک کلاس پسرانه پایه اول ابتدایی به صورت دیکته گفته شد و یکبار نیز توسط آزمونگر در طی دو مرحله به یک کلاس دخترانه و یک کلاس پسرانه دیگر پایه اول ابتدایی به صورت دیکته گفته شد. سپس خطاهای دیکته تعیین و مورد تجزیه و تحلیل آماری قرارگرفت.   یافته‌ها: نتایج نشان داد که خطاهای هجی کردن در مرحله اول و دوم اجرای آزمون توسط آزمونگر، هم در شکل نویسه (P=0/12) و هم در جایگاه نویسه (P=0/16) اختلاف معنی‌داری ندارد، در حالیکه در بین دو مرحله اجرای آزمون توسط آزمونگر و معلم در شکل نویسه تفاوت معنی‌دار مشاهده شد (P<0/01)، ولی در جایگاه نویسه با یکدیگر تفاوت معنی‌دار ندارند (P=0/12).   نتیجه‌گیری: نتایج بدست آمده گواه اعتبار و پایایی داخل آزمونگر آزمون در هر دو بخش شکل وجایگاه نویسه است.اما آزمون فقط در بخش جایگاه نویسه دارای اعتبار بین آزمونگر است.

Title: مقایسه تأثیر بار خارجی بر میزان انحنای کمر در زنان مبتلا به کمردرد مزمن با زنان سالم

http://rehabilitationj.uswr.ac.ir/article-1-200-fa.html

هدف: انحنای کمر یکی از مهمترین مشخصات پ ا سچر و حرکت بدن می‌باشد و در فهم بهتر مشکلات کمر درد و چگونگی ایجاد آن با اهمیت است. هدف از این مطالعه بررسی تأثیر بار خارجی و وضعیت تنه برمیزان انحنای کمر در زنان مبتلا به کمردرد مزمن در حالت استاتیک است.   روش بررسی: این تحقیق یک مطالعه مداخله‌ای شبه تجربی از نوع مورد - شاهدی است که بر روی 10 زن مبتلا به کمردرد مکانیکی مزمن و10 زن سالم به عنوان گروه کنترل و با استفاده از نمونه‌گیری ساده و غیر تصادفی انجام شد. با استفاده از دو حسگر شیب سنج الکترولیتی، میزان انحنای کمر افراد در شش فعالیت استاتیکی با سه سطح بار خارجی (صفر، شش و دوازده کیلوگرم) و دو وضعیت تنه (نوترال و خمیده 30 درجه) مورد بررسی قرار گرفت.   اطلاعات بدست آمده با استفاده از آزمونهای آماری کولموگراف – اسمیرنوف، تی مستقل و اندازه‌گیرهای مکرر (آنووا) مورد ارزیابی قرارگرفتند.    یافته‌ها: نتایج نشان داد که میزان لوردوز کمر در افراد مبتلا به کمردرد در دو وضعیتی که فرد بار 6 و 12 کیلوگرمی را با تنه نوترال حفظ کرده بود، بیشتر از افراد سالم است (P<0/05).   نتیجه‌گیری: افزایش لوردوز کمر در افراد مبتلا به کمردرد در حالت ایستاده و حین حفظ بار که در اثر عوامل مختلفی رخ می‌دهد، مخالف حالتی است که در افراد سالم وجود دارد و این افزایش انحنا که بصورت تطابق جبرانی به دلایلی از جمله کاستن درد و ترس از درد می‌باشد، همانند چرخه‌ای معیوب خود باعث افزایش کمردرد به دلیل نیروهای نامتناسب وارده به کمر در درازمدت می‌شود.

Title: بررسی شیوع جهش‌های ژن 2GJB در اقوام ایرانی

http://rehabilitationj.uswr.ac.ir/article-1-201-fa.html

هدف: ناشنوایی ارثی یک اختلال شایع است و هنگامی که به صورت الگوی وراثتی اتوزومی مغلوب به ارث می‌رسد، بعنوان یک پدیده منحصر به فرد بروز می‌کند. برخلاف هتروژنیتی زیاد در ناشنوایی، جهش‌ها در ژن «2 GJB » شایعترین علت ناشنوایی مادرزادی شدید تا عمیق در بسیاری از جوامع می‌باشد. در این مطالعه به بررسی جهش‌های ژن 2 GJB و یک حذف در ژن 6 GJB در ناشنوایی غیر سندرمی اتوزومی مغلوب در ایران پرداخته شد.   روش بررسی: در این مطالعه توصیفی مقطعی 1605 فرد مبتلا از 1605 خانواده با ناشنوایی غیرسندرمی با الگوی وراثتی مغلوب اتوزومی مورد بررسی قرارگرفتند. جهت انجام مراحل مختلف تحقیق، بعد از کسب رضایت‌نامه از افراد، تست ناشنوایی و آزمایشات بالینی، همراه با گرفتن cc 10 از خون محیطی بعنوان نمونه ای برای استخراج DNA انجام شد. پس از بررسی جهش delG 35 نمونه‌های منفی و هتروزیگوت از نظر این جهش، برای بررسی جهش‌های دیگر GJB2 به خارج از کشور فرستاده شد.   یافته‌ها: در 243 خانواده (%1/15) جهش در ژن 2 GJB عامل بروز ناشنوایی تشخیص داده شد. نتیجه‌گیری: تنوع جغرافیایی در فرکانس اللی جهش delG 35 در ایران نتایج قابل توجهی در مقایسه با سایر مطالعات در اروپا و نیز کشورهای همسایه داشته و حذف (1830 S 12 D - 6 ∆(GJB در هیچ فردی مشاهده نشد.

Title: بررسی تأثیر افزودن ماساژسوئدی به کاردرمانی بر تون عضلانی کودکان مبتلا به فلج مغزی اسپاستیک

http://rehabilitationj.uswr.ac.ir/article-1-202-fa.html

هدف : فلج مغزی به صورت اختلال غیر پیشرونده در وضعیت وحرکت بدن می‌باشد که دراثر علل مختلفی ایجاد می‌شود. اقدامات درمانی وتوانبخشی مختلفی در این کودکان ازجمله ماساژ به کار رفته است. تحقیق حاضر به منظور بررسی تأثیر افزودن درمان ماساژ به کاردرمانی در بهبود تون عضلانی کودکان مبتلا به فلج مغزی اسپاستیک انجام شد.   روش بررسی: در این کارآزمایی بالینی دو سویه کور نمونه‌ها ازمیان کودکان مبتلا به فلج مغزی مراجعه کننده به مراکز توانبخشی وابسته به دانشگاه علوم بهزیستی و توانبخشی جامعه در دسترس، به روش نمونه‌گیری مبتنی بر هدف و دارا بودن شرایط ورود به مطالعه انتخاب و بطور تصادفی به 2 گروه کنترل ومداخله تقسیم شدند.روش استاندارد توانبخشی درطی 3 ماه برای هردو گروه بطور یکسان اجرا شد و گروه مداخله قبل از کاردرمانی 30 دقیقه ماساژ نیز دریافت نمودند. تون عضلانی قبل وپس از مداخله بر اساس آزمون اشورث ارزیابی گردید. تجزیه و تحلیل داده‌ها با آزمونهای آماری پارامتری (تی زوجی و تی مستقل) و غیر پارامتری (ویلکاکسون و من ویتنی) صورت گرفت.   یافته‌ها: 13 نفر درگروه مداخله و 14 نفر در گروه کنترل مورد بررسی قرار گرفتند. میانگین سن در گروه مداخله 49/5 ماه و در گروه کنترل 42/1 ماه بود. تون اندام‌های فوقانی و تحتانی و عضلات تنه و گردن، قبل و پس از درمان در گروههای مداخله و کنترل اختلاف معناداری نداشت (P>0/05).   نتیجه‌گیری: براساس نتایج این تحقیق افزودن ماساژ به کاردرمانی درکودکان مبتلا به فلج مغزی اسپاستیک، بر تون عضلانی آنان تأثیری ندارد، ولی باتوجه به ذکر چنین تأثیری دربرخی مقالات و ازطرفی محدودیتهای اجرایی مختلف درانجام این تحقیق، نمی‌توان وجود اثر مثبت این روش را در بهبود تواناییهای حرکتی این کودکان رد کرد.

Title: بررسی نشانگان متقاطع لگنی در بیماران مبتلا به کمردرد مزمن غیر اختصاصی

http://rehabilitationj.uswr.ac.ir/article-1-203-fa.html

هدف: بررسی ارتباط نشانگان عدم تعادل عضلانی ناحیه کمری – لگنی (نشانگان متقاطع لگنی) که شامل ترکیب کوتاهی عضلات وضعیتی (Postural) همراه با ضعف عضلات فازیک این ناحیه است، با قوس کمری و کمردرد مزمن هدف این مطالعه می‌باشد.   روش بررسی: این تحقیق بصورت مقطعی‌ - مقایسه‌ای و از نوع‌ مورد - شاهدی انجام‌ شد. به طریق نمونه‌گیری‌ ساده و بر اساس معیارهای مورد نظر 600 نفر (300 فرد سالم-300 بیمار کمردردی) در این تحقیق مورد بررسی قرار گرفته و قوس کمر، قدرت عضلات شکم و اکستانسور هیپ و طول عضلات ایلیوپسواس و خلف کمر در آنها اندازه‌گیری شد. با استفاده از آزمون Receiver Operating Characteristic (ROC) curve افراد به گروههای دارای ترکیبی از ضعف یا کوتاهی عضلات مطرح شده در نشانگان متقاطع لگنی یا بدون آنها در هر دو گروه تقسیم شدند و ارتباط این نشانگان با اندازه قوس کمر و کمردرد در دو گروه مورد مقایسه قرار گرفت. جهت تجزیه و تحلیل‌های آماری از آزمونهای ICC ، کولموگراف – اسمیرنوف، تی مستقل و آنالیز واریانس استفاده شد.   یافته‌ها: هیچگونه اختلاف معنی‌داری بین قوس کمری افراد سالم و دارای اختلالات عضلانی نشانگان متقاطع لگنی (P=0/38) و همچنین بین قوس کمری افراد سالم و بیماران کمردردی دارای اختلالات عضلانی این نشانگان (P=0/62) وجود نداشت. همچنین اختلاف معنی‌داری بین قوس کمری افراد سالم و بیماران مبتلا به کمردرد نیز مشاهده نشد (P=0/25).   نتیجه‌گیری: این نتایج تئوری نشانگان متقاطع لگنی که در آن عنوان شده ترکیب اختلالات عضلانی خاصی باعث افزایش قوس کمری و کمردرد می‌شود را تأیید نمی‌کند. اگرچه ارتباط معنی‌داری بین اختلالات عضلانی با کمردرد وجود دارد، اما بنظر می‌رسد این ارتباط از طریق تغییر در اندازه قوس کمر مطابق آنچه در این نشانگان ذکر شده نیست.

Title: تأثیر شعردرمانی گروهی بر وضعیت روانی بیماران اسکیزوفرنیک

http://rehabilitationj.uswr.ac.ir/article-1-204-fa.html

هدف: اسکیزوفرنیا یکی از اختلالات پیچیده روانپزشکی می‌باشد. گرچه داروهای ضد روانپریشی تکیه‌گاه اصلی درمان اسکیزوفرنیا به شمار می‌روند، ولی مداخلات روانشناختی نیز در بهبود نشانه‌های بالینی مؤثر هستند.شعردرمانی نیز به عنوان یکی از این مداخلات، ممکن است در بهبود جنبه‌های روانی - اجتماعی اسکیزوفرنیا مؤثر باشد.هدف این مطالعه بررسی تأثیر شعردرمانی گروهی بروضعیت روانی بیماران اسکیزوفرنیک می‌باشد.   روش بررسی: این پژوهش یک مطالعه تجربی است. در این مطالعه تعداد 29 بیمار اسکیزوفرنیک به روش مبتنی برهدف از مرکز آموزشی درمانی روانپزشکی رازی انتخاب و به صورت تصادفی تعادلی در دو گروه مداخله (14بیمار) و شاهد (15بیمار) قرار گرفتند. برای گروه مداخله 12جلسه یک ساعته شعردرمانی، هفته‌ای دو بار به مدت 6هفته برگزار شد. ابزار گردآوری داده‌ها شامل پرسشنامه اطلاعات دموگرافیک و مقیاس مختصر ارزیابی روانپزشکی (BPRS) بودند. تجزیه و تحلیل آماری به وسیله آزمون‌های آماری تی زوجی و تی مستقل انجام شد.   یافته‌ها: نتایج تفاوت معناداری را در گروه مداخله قبل و بعد از مداخله شعردرمانی نشان داد (P<0/001). این در حالی است که در گروه شاهد تفاوت معناداری بین میانگین‌های قبل و بعد وجود نداشت (P=0/774). همچنین پس از شعردرمانی بین دو گروه تفاوت معناداری از لحاظ آماری وجود داشت (P<0/05)، در حالی که قبل از شعردرمانی گروهی، تفاوت معناداری بین گروه مداخله و شاهد وجود نداشت (P=0/812).   نتیجه‌گیری: یافته‌های این مطالعه نشان داد که مداخله شعردرمانی بر بهبود وضعیت روانی بیماران اسکیزوفرنیک مؤثر است.

Title: مقایسه تأثیر گُوِه داخلی خلف پا با گُوِه خارجی قدام پا بر بهبود میزان فعالیتهای روزمره و پرداختن به فعالیتهای ورزشی و سرگرمی و درد دربیماران مبتلا به پلانتار فاشیاتیس

http://rehabilitationj.uswr.ac.ir/article-1-205-fa.html

هدف: پلانتار فاشیاتیس یکی از شایعترین اختلالات در ناحیه پا می‌باشد که سبب بروز درد در سمت داخلی لبه قدامی استخوان پاشنه می‌شود. هدف این مطالعه مقایسه تأثیر گوه داخلی خلف پا با گوه خارجی قدام پا بر بهبود میزان فعالیتهای روزمره و پرداختن به فعالیتهای ورزشی و سرگرمی و کاهش درد در بیماران مبتلا به پلانتار فاشیاتیس می‌باشد.   روش بررسی: این مطالعه از نوع شبه تجربی می‌باشد. 30 بیمار مبتلا به پلانتار فاشیاتیس شامل 12 مرد و 18 زن با 36 پاشنه دردناک با دامنه سنی 40-30 سال از بین نمونه‌های در دسترس، با روش غیر احتمالی ساده انتخاب گردیده و در دو گروه 15 نفری به طور تصادفی قرار گرفتند. به گروه اول گوه داخلی خلف پا و به گروه دوم گوه خارجی قدام پا داده شد. متغیرهای مورد بررسی شامل میزان فعالیت روزمره، فعالیتهای ورزشی و سرگرمی، درد و طول قدم می‌باشند که در 3 مرحله اندازه‌‌گیری شدند. ابزار مورد استفاده در این تحقیق پرسشنامه FAOS بود. 3 مرحله اندازه‌گیری شامل یک مرحله قبل از مداخله و دو مرحله دیگر شامل 2 و 4 هفته بعد از مداخله بود.   ضریب پایایی (آلفای کرونباخ) پرسشنامه در تمامی زیر مجموعه‌ها بیشتر از 0/71 بود. ضریب همبستگی آزمون-بازآزمون بیماران در تمام زیرمجموعه‌های پرسشنامه بیشتر از 0/80 بود. از آزمونهای تی مستقل، اسمیرونوف کولموگروف، آلفای کرونباخ و ضریب همبستگی برای تجزیه و تحلیل داده‌ها استفاده گردید.   یافته‌ها: در مقایسه دو گروه در هر دو مرحله پیگیری، نتایج در گروه با گوه داخلی خلف پا در مورد بهبود میزان فعالیتهای روزمره، فعالیتهای سرگرمی و ورزشی و کاهش درد نسبت به گروه با گوه خارجی قدام پا بهتر و اختلاف آنها معنادار بود (P<0/05). ولی بین طول قدم در دو گروه اختلاف معناداری دیده نشد (در مرحله اول پیگیری P=0/518 و در مرحله دوم پیگیری P=0/502).   نتیجه‌گیری: بر اساس این نتایج، گوه داخلی خلف پا نسبت به گوه خارجی قدام پا تأثیر بهتری در بهبود فعالیتهای روزمره زندگی و پرداختن به فعالیتهای ورزشی و سرگرمی و کاهش درد بیماران پلانتار فاشیاتیس داشته، ولی بر افزایش طول قدم نسبت به گوه خارجی قدام پا تأثیر بهتری ندارد.

Title: عوارض چشمی دیررس گاز خردل در 500 نفر از مجروحین شیمیایی جنگ تحمیلی

http://rehabilitationj.uswr.ac.ir/article-1-206-fa.html

هدف: با عنایت به کاربرد وسیع گازهای شیمیایی به خصوص گازخردل درجنگ تحمیلی عراق علیه ایران و عوارض ناشی ازآن، این تحقیق به منظور بررسی میزان ضایعات چشمی دیررس در مجروحین شیمیایی جنگ تحمیلی انجام گرفت.   روش بررسی: این پژوهش به روش تحلیلی انجام شد. از بین مجروحین شیمیایی 13 استان کشور که به کمیسیون پزشکی تعیین درصد بنیاد معرفی شده بودند، نمونه‌ای به تعداد 500 نفر با معیارهای سن بین 55-35 سال، مدت زمان مصدومیت با گاز خردل بیش از 18 سال، عدم وجود عارضه چشمی غیر جنگی، صورت سانحه معتبر شیمیایی و مدرک درمانی همزمان با مصدومیت با گاز خردل مورد تأیید بنیاد، به صورت هدفمند انتخاب شدند. شدت ضایعات چشمی دیررس نمونه‌ها تعیین و ارتباط آنها با متغیر‌های دفعات مجروحیت، مدت زمان بستری، استفاده از داروهای چشمی، مصرف سیگار و استفاده از وسایل حفاظتی بررسی گردید. داده‌های حاصل با استفاده از ضریب همبستگی اسپیرمن و آزمون خی دو مورد تجزیه و تحلیل قرار گرفت.   یافته‌ها: فراوانی ضایعات چشمی خفیف %80، متوسط %2/13 و شدید %8/6 بود. %2/82% نفرات یکبار، %2/12 دوبار، %8/1 سه بار و %8/3 آنها بیش از سه بار مجروحیت داشته و رابطه معنی‌داری بین دفعات تماس با گاز خردل و شدت ضایعات چشمی وجود داشت (P<0/05) . مدت زمان بستری رابطه معنی‌داری (P<0/050) با شدت ضایعات چشمی نشان ‌داد. %8/89‌ از مجروحین شیمیایی سابقه مصرف داروی چشمی داشته و رابطه معنی‌داری بین شدت ضایعات چشمی و مصرف دارو وجود داشت (P>0/01) .   نتیجه‌گیری: عوارض چشمی دیررس در تمامی مجروحین شیمیایی با گاز خردل وجود داشته و نوع خفیف آن به مراتب فراوان تر از سایر درجات است. افزایش دفعات مجروحیت شیمیایی با گاز خردل موجب افزایش نیاز به داروهای چشمی، مدت زمان بستری بیشتر و شدت بیشتر ضایعات چشمی می‌گردد.

Title: مقایسه باورهای غیر منطقی مادران دارای فرزند معلول ذهنی شدید یا عمیق با مادران دارای فرزند عادی

http://rehabilitationj.uswr.ac.ir/article-1-220-fa.html

هدف: تحقیق حاضر به منظور مقایسه باورهای غیرمنطقی مادران دارای فرزند معلول ذهنی شدید یا عمیق با مادران دارای فرزند عادی 14-6 ساله شهر تهران انجام گرفت.   روش بررسی: مطالعه حاضر به صورت تحلیلی، مقطعی و مقایسه‌ای از نوع مورد- شاهدی بوده و 80 نفر از مادران دارای فرزند معلول ذهنی شدید یا عمیق مراجعه کننده به مراکز خدمات بهزیستی تهران بصورت تصادفی منظم از دو مرکز توانبخشی شهر تهران و 80 نفر از مادران دارای فرزند عادی جهت همتا سازی به عنوان گروه شاهد از نواحی شرق، غرب، جنوب، شمال و مرکز تهران از طریق نمونه گیری خوشه‌ای چند مرحله‌ای و با لحاظ جور بودن در چهار متغیر ؛ سن مادران، سطح تحصیلات، منطقه محل سکونت وتعداد فرزندان انتخاب شدند و به پرسشنامه باورهای غیرمنطقی جونز( IBT ) پاسخ دادند.   تجزیه و تحلیل داده‌ها با استفاده از آمار توصیفی و استنباطی (تی مستقل، یو مان ویتنی، مجذور کای و آزمون دقیق فیشر) انجام شد.   یافته‌ها: نتایج نشان داد که تفاوت معناداری بین باورهای غیرمنطقی به طور کلی و باورهای غیر منطقی سرزنش کردن خود، واکنش با درماندگی به ناکامی، نگرانی زیاد توأم با اضطراب، اجتناب از مشکل، وابستگی و کمال‌گرایی بین دو گروه وجود دارد (05/0 p< ). بین مادران دارای فرزند معلول ذهنی دختر با پسر تفاوت معناداری مشاهده نشد(314/0= P ). بین دو گروه مادران در چهار باور توقع تأیید از دیگران (737/0= P )، انتظارات بیش از حد از خود (126/0= P )، بی مسئولیتی عاطفی(283/0= P ) و درماندگی برای تغییر (283/0= P ) تفاوت معنی‌داری مشاهده نشد.   نتیجه‌گیری: باورهای غیرمنطقی مادران دارای فرزند معلول ذهنی عمیق یا شدید بطور کلی و در اکثر خرده مقیاسهای آن بیشتر از مادران دارای فرزند عادی است و فقط باورهای توقع تأیید از دیگران، انتظارات بیش از حد از خود، بی‌مسئولیتی عاطفی و درماندگی برای تغییر در دو گروه تفاوتی ندارد. همچنین جنسیت فرزند معلول تأثیری در باورهای غیر منطقی مادران ندارد.

Title: توان‌بخشی درایران باستان

http://rehabilitationj.uswr.ac.ir/article-1-208-fa.html

هدف:‌ ناتوانی و معلولیت به مفهوم امروزین خود، در جهان باستان و کتابهای پزشکی و قدیمی کمتر به چشم می‌خورد، اما ردپای «درمان بخشی» و «توانمندسازی» در تاریخ بیشتر سرزمین‌های کهن مشهود است که با اعتقادات و باورهای دینی همراه بوده است.   هدف از پژوهش حاضر، بررسی آثار و اسناد تاریخی معتبر باقی مانده از دوران باستان در خصوص توانبخشی است تا بتوانیم سهم تمدن ایرانی را در روند شکل‌گیری «توانبخشی» و «توانمندسازی» در جهان امروز بر پایه مستندات علمی به دست آوریم.   روش بررسی:‌ این تحقیق از روش پژوهش کیفی از طریق مروری در اسناد تاریخی می‌باشد که پس از تبیین هدف و پی‌ریزی فرضیه‌های مد نظر به جمع‌آوری اطلاعات دقیق از منابع معتبر با روش تحقیقات کیفی پرداخته است.   یافته‌ها:‌ در ایران باستان اهورامزدا سرچشمه همه نیکی‌ها و اهریمن منشاء همه ‌پلیدی‌هاست. درد و رنج و بیماری از وسوسه‌های اهریمن است. تمدن ایرانیان یکی از نخستین تمدنهایی بوده که از روش‌های علمی و خواص گیاهان دارویی برای درمان بخشی و توانمندسازی استفاده می‌کرده است. دانشگاه گندی شاپور نخستین دانشگاه پزشکی بوده که به امر فرهنگی و توانبخشی می‌پرداخته و نیز مکان‌هایی در آن زمان بوجود آمده که به امر نگهداری از معلولین و محرومین اختصاص داشته است.   نتیجه‌گیری: زمانی که غرب و سایر ملل درگیر جهل و نادانی بوده و از ترس بیماری به خود می‌لرزیدند و معلولان را به دریا می‌انداختند، ایران مهد تمدن و مرکز بزرگترین دانشگاه پزشکی بود. از آنجا که اسناد معتبر و به جای مانده بسیار محدود و مطالب پراکنده می‌باشد، لذا هر عبارت و نکته حاصله در این پژوهش خود بیانگر مطالب مهم و غیرقابل اغماض می‌باشد که شرح و تفسیر آن به عهده پژوهشگران و خوانندگان این مقاله می‌باشد.
